# Perspective on Cancer Control: Whither the Tobacco Endgame for Canada?

**DOI:** 10.3390/curroncol29030168

**Published:** 2022-03-18

**Authors:** Elizabeth A. Eisenhauer, Robert Schwartz, Rob Cunningham, Les Hagen, Geoffrey T. Fong, Cynthia Callard, Michael Chaiton, Andrew Pipe

**Affiliations:** 1 Department of Oncology, Queen’s University, Kingston, ON K7L 3N6, Canada; 2 Ontario Tobacco Research Unit, University of Toronto, Toronto, ON M5T 3M7, Canada; robert.schwartz@utoronto.ca (R.S.); michael.chaiton@camh.ca (M.C.); 3 Canadian Cancer Society, Ottawa, ON K1P 5G3, Canada; rob.cunningham@cancer.ca; 4 Action on Smoking & Health, Edmonton, AB T5J 1V9, Canada; hagen@ash.ca; 5 School of Public Health and Health Systems, University of Waterloo, Waterloo, ON N2J 4B6, Canada; geoffrey.fong@uwaterloo.ca; 6 Ontario Institute for Cancer Research, Toronto, ON M5G 0A3, Canada; 7 Physicians for a Smoke-Free Canada, Ottawa, ON K1Y 0S9, Canada; ccallard@smoke-free.ca; 8 University of Ottawa Heart Institute, University of Ottawa, Ottawa, ON K1Y 4W7, Canada; apipe@ottawaheart.ca

**Keywords:** tobacco control, Tobacco Endgame, Canada

## Abstract

Aims: In 2014, in response to evidence that Canada’s tobacco use would lead, inexorably, to substantial morbidity and mortality for the foreseeable future, a group of experts convened to consider the development of a “Tobacco Endgame” for Canada. The “Tobacco Endgame” defines a time frame in which to eliminate structural, political, and social dynamics that sustain tobacco use, leading to improved population health. Strategies: A series of Background Papers describing possible measures that could contribute to the creation of a comprehensive endgame strategy for Canada was prepared in advance of the National Tobacco Endgame Summit hosted at Queen’s University in 2016. At the summit, agreement was reached to work together to achieve <5% tobacco use by 2035 (<5 by ’35). A report of the proceedings was shared widely. Achievements: Progress since 2016 has been mixed. The Summit report was followed by a national forum convened by Health Canada in March 2017, and in 2018, the Canadian Government adopted “<5 × ’35” tobacco use target in a renewed Canadian tobacco reduction strategy. Tobacco use has declined in the last 5 years, but at a rate slower than that which will be needed to achieve the <5 by ’35 goal. There remain > 5 million smokers in Canada, signaling that smoking-related diseases will continue to be an enormous health burden. Furthermore, the landscape of new products (e-cigarettes and cannabis) has created additional risks and opportunities. Future directions: A bold, reinvigorated tobacco control strategy is needed that significantly advances *ongoing* policy developments, including full implementation of the key demand-reduction policies of the WHO Framework Convention on Tobacco Control. Formidable, new *disruptive* policies and regulations will be needed to achieve Canada’s Endgame goal.

## 1. Introduction: The Start of the Tobacco Endgame Discussion in Canada

In 2014, it was clear that, despite decades of effort and the impact of tobacco-control measures on Canada’s smoking rates, tobacco use remained the leading preventable cause of disease and death in Canada. More than 500,000 person years of life were being lost annually to smoking-related diseases, including various cancers as well as lung and cardiovascular diseases [1]; at that time (2013), the current smoking prevalence was 19% of the population [2], indicating that the pandemic of tobacco-related illness would continue for many years. Furthermore, modeling of Ontario data by the Ontario Tobacco Research Unit indicated that, even with full implementation of the MPOWER measures of the WHO Framework Convention on Tobacco Control (FCTC), smoking prevalence in Canada would remain above 10% for at least another decade, with the total number of smoking-related deaths continuing to rise [3].

These observations galvanized a small group of clinicians, tobacco control experts, advocates, and scientists, initially supported by Queen’s University, to come together to consider what new, transformative measures would be needed for Canada to achieve the “Tobacco Endgame”. According to Dr. Ruth Malone, an early proponent, the Tobacco Endgame consists of “Novel initiatives designed to change and eliminate permanently the structural, political, and social dynamics that sustain the tobacco epidemic in order to end it, within a specific time frame” [4]. Thus, an Endgame strategy requires both a tobacco-prevalence target and a timepoint for achieving that target.

A Steering Committee was formed to develop a pan-Canadian Summit at which a Tobacco Endgame for Canada could be debated, mutual goals developed, and an examination of the scope of the necessary transformative measures undertaken. The Steering Committee proposed a target of less than 5% tobacco use by 2035 (<5 by ’35) as Canada’s endgame goal. It was agreed that that this goal could only be achieved through a set of measures that would collectively limit the number of new smokers and increase the number of current smokers who would quit. Focusing on only one of these subgoals would not achieve the <5 by ’35 goal. Four action groups were created to prepare a comprehensive pre-Summit background paper to identify and describe possible measures that could contribute to the creation of a comprehensive endgame strategy for Canada.

The Background document (https://www.otru.org/wp-content/uploads/2021/09/EndgameSummit-Backgroundpaper-Aug-30-2016.pdf; accessed 13 February 2022) consisted of 41 potential Tobacco Endgame measures. At the Summit hosted by Queen’s University in September 2016, 85 attendees from across Canada, representing government, non-government, clinical, and public health perspectives, assembled to examine and debate these measures. There was unanimous agreement on the need to develop an endgame strategy addressing the use of commercial tobacco in Canada to achieve the <5 by ’35 goal. Major areas for action were identified, as shown in Table 1.

At the time of the Summit, e-cigarettes were emerging as a new product and, although scant data were available regarding their putative benefits (in terms of harm reduction or as cessation aids for smokers) or potential harms (e.g., in creating new nicotine addiction in youth or never-smokers, or in undermining cessation) cautions were expressed regarding the need for their appropriate, thoughtful regulation.

The Summit concluded with a commitment to continue to work together to advance the key measures identified as central to the development of an Endgame. A Summit report was created for wide circulation (https://www.otru.org/wp-content/uploads/2021/09/Tobacco-Endgame-Summit-Summary-Report.pdf; accessed 13 February 2022). The Summit report was followed by a national forum convened by Health Canada in March 2017, and the subsequent adoption of the “<5 × ’35” tobacco use target by the federal government in a renewed Canadian tobacco-reduction strategy in May 2018.

This paper summarizes a number of topics discussed at a recent Canada-wide webinar hosted by the Ontario Tobacco Research Unit to mark the fifth anniversary of the Summit. The topic, “*Whither Canada’s Tobacco Endgame*?”, provided an opportunity to review the tobacco control gains made—and opportunities missed—since 2016, to document progress made towards the Endgame Goal, and to re-invigorate the tobacco-control community as it works to achieve this goal.

## 2. After the Summit—What Has Been Achieved in Canada?

### 2.1. Changes in Population Tobacco and Nicotine Behaviours since 2016

**Figure 1:**
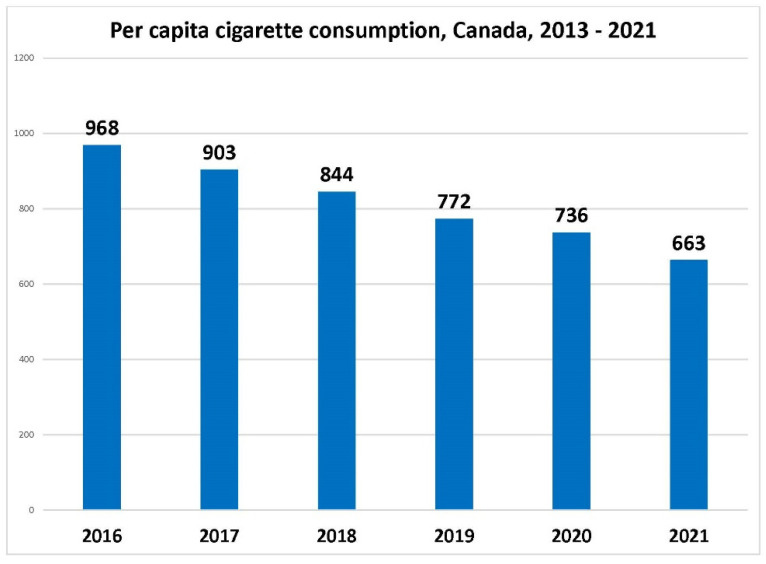
Per capita cigarette consumption, Canada, 2016–2021 (first 9 months). Sources: Annual cigarette and roll-your-own tobacco sales, 2016–2020 inclusive, from Health Canada, based on tobacco industry reports pursuant to Tobacco Reporting Regulation; sales volumes for 2021 based on a 9.3% decline for the Canadian market as reported by Philip Morris International; Canadian population data from Statistics Canada.

**Figure 2:**
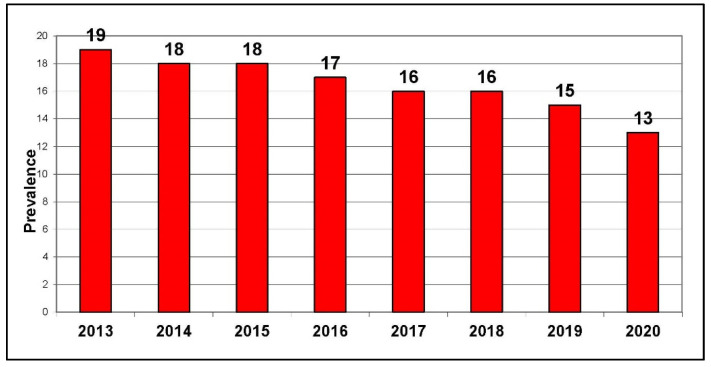
Current smoking prevalence (%) in Canada, age 12+, 2013–2020. Source: Canadian Community Health Survey.

As shown in Figure 1 and Figure 2, since 2016, there has been a continual decline in the number of cigarettes sold and in the number of smokers in Canada. The Canadian Community Health Survey in 2020 revealed there are still more than five million smokers in Canada [5], a decline in absolute numbers from 2000, reflecting both cessation success but also, sadly, deaths in smokers. Just over 60% of smokers are male [5]. Statistics Canada advises caution when using data from 2020, however, due to the changes in survey methods and in respondents during the COVID-19 pandemic. Nonetheless, millions of smokers in Canada remain, signaling that smoking-related diseases will continue to be an enormous, but largely preventable, health burden in Canada.

Since the 2016 summit, there has been a substantial change in the *use of e-cigarettes* or electronic nicotine delivery systems (ENDSs). Among high school students in Canada in grades 10–12, past 30-day e-cigarette use increased from 9% in the 2014–2015 school year, to 16% in 2016–2017, and to 29% in 2018–2019. For students in grades 7–9, the increase was from 3% in 2014–2015 to 5% in 2016–2017 to 11% in 2018–2019. In terms of total nicotine use from either smoking or using e-cigarettes in the past 30 days, among grade 10–12 students, the increase was from 16% in 2014–2015 to 31% in 2018–2019 [6].

Another metric being tracked is the *number of quit attempts* in current smokers, which has remained essentially unchanged for many years—about half of smokers report making a quit attempt, largely unassisted, at least once a year. Similarly, the quit “success rate” has been remarkably steady—typically hovering at around 2% of quit attempts (data derived from Chaiton et al. [7]).

### 2.2. Tobacco Control Policy Changes since the Summit

Although the endgame objective of less than 5% tobacco use by 2035 as a goal was endorsed by Canada’s federal government in May 2018, it has not yet been endorsed by any province or territory. A number of government measures have been adopted since 2016, but collectively, these measures are unlikely to realize the “<5 by ’35” goal because they have not been truly disruptive to the structural, political, and social dynamics that sustain the tobacco epidemic. The actions taken since 2016 are presented subsequently.

#### 2.2.1. Taxation and Price

**Figure 3:**
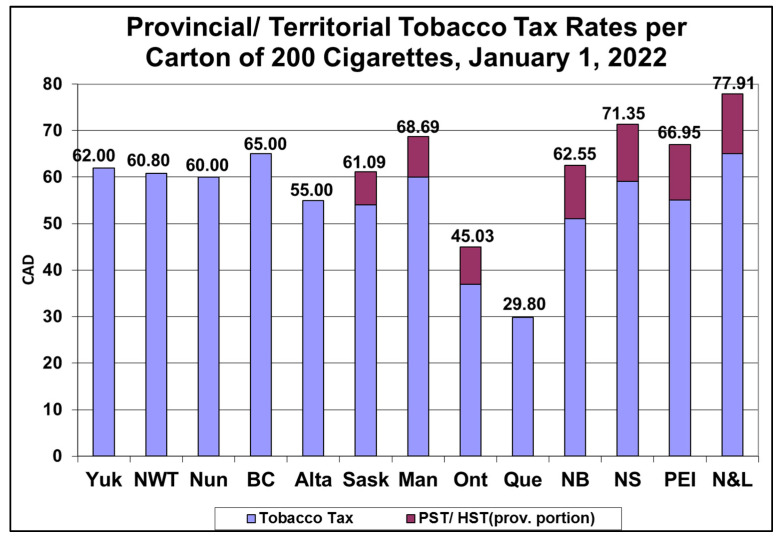
Provincial/territorial tobacco tax rates in CAD.

Federal tobacco tax rates have increased by CAD 8.05 per carton of 200 cigarettes between January 2017 and January 2022, and the majority of provinces/territories have also increased tobacco taxes. Federal tobacco taxes are now indexed annually to inflation. Provinces/territories have quite a variation in tax rates, with Ontario and Quebec—the two most populous provinces—having the lowest rates (Figure 3). Tobacco manufacturers have also increased their net-of-tax prices significantly, by CAD 23.20 per carton on average Canada-wide for 2014 to 2021 (first half) inclusive (Table 2). Overall, the price of purchasing a carton of cigarettes has increased substantially since 2014 (by CAD 37–57 per carton, depending on province/territory) (Table 2). Ironically, in most provinces/territories, the majority of total price increases have been imposed by the tobacco industry itself (Table 2). Price increases have long been recognized as an important factor influencing lower adult and youth prevalence and per capita consumption. Going forward, there are significant opportunities for further tobacco tax increases.

#### 2.2.2. Retail Packaging and Product

Canada implemented the world’s best plain packaging tobacco regulations in 2020, with implementation of the slide and shell package format in retail as of February 2022 [8]. These new requirements also regulate cigarette length and width (banning slims and 100 mm cigarettes).

#### 2.2.3. Flavoured Tobacco

At the time of the Summit, several provinces had banned flavoured tobacco including menthol; however, a national ban on menthol cigarettes was implemented for cigarettes in 2017 and all tobacco products in 2018 [9]. Depending on the province, some categories of flavoured tobacco products remain available for sale.

#### 2.2.4. Restrictions on Smoking

The past 5 years have seen some expansion of smoke-free laws and policies: for example, provinces/territories/municipalities have restricted smoking in more outdoor areas; Saskatchewan and Nunavut have adopted smoke-free social housing, joining Yukon; and, as of 2021, there are more than 100 smoke-free university/college campuses across Canada that are 100% smoke-free (compared with only 15 in 2016).

#### 2.2.5. Restrictions on Sales and Promotions

Since the Summit, a minimum sales age of 21 has been implemented in one jurisdiction in Canada: Prince Edward Island. In contrast, a national minimum age of 21 was implemented in the United States in December 2019, and the measure is now also included in the law of 30 states.

Nunavut has adopted legislation (awaiting proclamation) to prohibit lower prices based on quantity sold (as did the Northwest Territories), to prohibit manufacturer incentive promotions to retailers (e.g., bonuses for higher sales volumes, or for maintaining lower retail prices), and to prohibit manufacturers from giving lower prices to different retailers.

#### 2.2.6. Manufacturer Cost Recovery Fee

In the 2021 federal election campaign, the Liberal Party, the Conservative Party, and the NDP included a tobacco manufacturer cost recovery fee in their platforms. This would implement the “polluter pays” principle and require reimbursement from manufacturers for the CAD 66 million annual cost of the federal tobacco control strategy.

#### 2.2.7. Health Warnings

Health Canada is considering a new range of tobacco package health warnings, which have not been changed since 2012. As part of the 2018 consultation on new warnings, Health Canada included requiring a warning on the cigarette itself.

#### 2.2.8. E-Cigarettes

At the time of the 2016 Summit, nicotine-containing e-cigarettes (vaping products; ENDS—electronic nicotine delivery systems) were not yet legalized. This changed in May 2018, with the tobacco industry entering the Canadian e-cigarette market following legalization [10]. As many predicted, in the absence of effective regulation, youth and young adult vaping subsequently increased dramatically. In response to higher youth vaping, many governments have adopted additional e-cigarette measures, including taxation (four provinces and intended federally); increased federal promotion restrictions (some provinces/territories already had restrictions stronger than initial federal restrictions); maximum nicotine concentrations (federal and two provinces); flavour restrictions (three provinces and proposed federally); restrictions on the location of sale for flavoured products (three provinces); and package labelling requirements (federal and one province). All provinces/territories have legislation addressing sales to minors, retail display bans except in specialty vape stores, prohibiting e-cigarette use in places where smoking is banned, and prohibiting sales in places where tobacco sales are banned. Despite these developments, many e-cigarette regulatory measures recommended by the Council of Chief Medical Officers of Health remain unimplemented [11].

#### 2.2.9. Litigation against the Tobacco Industry

In March 2019, the tobacco industry suffered a major loss before the Quebec Court of Appeal, when this court upheld a Quebec Superior Court ruling in class action lawsuits and awarded CAD 13.5 billion in damages [12]. The industry responded by obtaining creditor protection, which put on hold the damage award as well as other lawsuits against the tobacco industry, including health care cost recovery lawsuits from all 10 provincial governments collectively seeking more than CAD 500 billion in damages. The granting of creditor protection prompted the beginning of confidential settlement negotiations between provinces and tobacco companies. These negotiations, which are ongoing, provide a one-time, historic opportunity to obtain significant measures in a settlement to reduce tobacco use, including restructuring of the tobacco industry and reforming its behaviour, and securing substantial long-term funding which could be deployed for tobacco control.

### 2.3. A Reality Check—Is “<5 by ’35” Achievable?

The answer to this question is mixed: there is good news and bad news

The good news: Canada has already achieved <5 by ’35 among a key target group: *youth*. National surveys, e.g., the Canadian Community Health Survey, 2020 [5], have revealed that overall smoking rates among youth aged 12 to 17 (grades 7 to 12) are below 5%. This achievement is no small feat, and has resulted from decades of concerted efforts by health organizations, advocates, practitioners, academics, governments, and educators. Nevertheless, other population groups have demonstrated much higher prevalence. In particular, the groups identified as priorities for action in 2018 included Indigenous peoples, LGBTQ+ persons, young adult males, workers in certain industries, people living in poverty, and those suffering with mood or anxiety disorders. In Canada, about 80% of smokers are affected one or more of these conditions [13].

The challenge now is not only to address the needs of priority groups, but to maintain this trajectory among youth, especially given the high numbers of underage youth now vaping (over 400,000), as described earlier. Data indicate that youth who vape are three times more likely to start smoking [14], making the rise in vaping in this demographic a significant concern. The increase in youth vaping is a reflection of government failure (despite warnings from the health community), to appropriately regulate these products and their marketing, resulting in extensive promotional exposure to youth.

Another potential blow to continued reduction in tobacco use has resulted from the legalization of cannabis in 2018. Canada is one of the few jurisdictions in the world to allow the widespread *public* consumption of cannabis. The federal government deferred the control of public consumption to the provinces, and some have done a better job than others in restricting cannabis use. Thus “smoking” in public has been re-normalized to some degree. Furthermore some 30% of those who smoke cannabis mix it with tobacco, and this is even higher amongst young people.

Although we have witnessed success in reducing population-level tobacco use, particularly among youth, the potential achievement of the “<5 by ’35” goal remains in question. It is essential, particularly in the face of other public health challenges (e.g., COVID-19 and climate change), that Endgame measures are constantly and consistently presented to governments and policy makers to ensure that this important public health issue is not ignored. This work requires concerted efforts from health charities, health professions, public health leaders, tobacco control organizations and advocates. In this respect, the permanent closure of three of Canada’s leading tobacco control organizations in recent years (the Non-Smokers’ Rights Association, the Canadian Council for Tobacco Control, and the Ontario Campaign for Action on Tobacco) due to lack of funding support has been a major blow to the Endgame effort.

The need for adequate and sustainable funding for tobacco control has never been greater if tobacco Endgame goals are to be achieved. Unfortunately, Canada’s tobacco reduction strategy is currently not aligned with its stated <5 by ’35 objective. For this problem to be corrected, benchmarks, milestones, and tangible action plans must be created by federal and provincial governments [15]. Optimistic guidance documents are not enough. There remains an unmet need for dynamic, robust initiatives on the part of Ministries of Health at every level to address Canada’s leading cause of preventable disease, disability and death. We have much to learn from other nations with well designed Endgame plans (see below). It remains possible for Canada to achieve <5 × ’35—but doing so will require applying every effective tool that we have at our disposal: higher taxes, more smoking bans, age 21 laws, more mass media campaigns, anti-contraband measures, greater restraints on the tobacco and vaping industries, more help for smokers who want to quit, and securing adequate and sustainable funding for tobacco control. Additionally, and perhaps most importantly, to address the root cause of the tobacco pandemic: a rogue industry which has for too long escaped adequate regulation and control.

### 2.4. Endgame Developments Globally

Countries engaged in Tobacco Endgame discussions fall into two main groupings: those proposing primarily *incremental* approaches versus those proposing more *disruptive* measures.

Incremental measures include stronger implementation of the key demand-reduction policies of the FCTC (known also as the WHO’s MPOWER measures): significant increases in tobacco taxes, comprehensive smoke-free laws, large graphic warnings, media campaigns, bans on tobacco advertising, promotion, and sponsorship, and support for cessation. They also include extensions of these policies, including plain packaging and Tobacco 21 (increasing the legal age of sales to 21 years). Although research has shown that countries implementing a greater number of these basic FCTC policies at the highest level experienced a substantially greater reduction in smoking prevalence in the first decade of the FCTC (2005–2015) [16], the overall level of implementation of these policies was very low (1.04 out of 5), highlighting the enormous unfulfilled potential of the FCTC because of poor implementation [16]. The FCTC Conference of the Parties has now shifted to prioritizing stronger implementation of the FCTC through its Global Strategy to Accelerate Tobacco Control [17] in an effort to strengthen and accelerate implementation, focusing on these key policies.

A number of high-income countries have already implemented a number of these basic policies; thus, there has been a need to explore other, disruptive measures to achieve Endgame goals. These include:

- Linking approaches to tobacco control to broader objectives such as tackling health inequities;
- Substantial strengthening of supply measures, such as dramatically reducing the number of tobacco retailers and more substantial regulation of the industry itself.

#### Product Regulation Changes Designed to Reduce Appeal and Addictiveness of Tobacco Products

In addition to banning flavours (which Canada has already implemented in cigarettes), potential regulations for the product itself that have been discussed include restricting additives, reducing nicotine contents in cigarettes to minimally addictive or to non-addictive levels, and banning filters.

An increasing number of jurisdictions globally are adopting endgame prevalence targets, including Denmark, England, the European Union, Finland, France, Ireland, New Zealand, Scotland, and Sweden. The approaches to achieving endgame targets vary. Finland’s objective relates to all nicotine use, not just tobacco use. [18] The Netherlands has adopted legislation to greatly reduce retail access to tobacco products, a strategy that was implemented one decade ago in Hungary.

New Zealand announced an action plan on 9 December 2021 designed to achieve a goal that by 2025, less than 5% of New Zealanders will be smokers, although legislation and regulations have not yet been adopted [19]. Their “Smokefree Aotearoa 2025” action plan includes “disruptive” measures that have long been discussed, including: (1) creating a “smokefree generation” policy, which defines who can legally be sold tobacco products by year of birth (only those born before 2008) rather than by age, which thus annually increments the age cohorts that cannot legally be sold tobacco by one year; (2) reducing addictiveness and appeal by requiring severe limits on nicotine below addictive levels (New Zealand as an island country and would be less susceptible to an increased illicit market than other countries, including Canada, would have), and to consider prohibiting filters and other design innovations that are known to increase attractiveness, decrease harshness, and to increase consumer misperceptions that such cigarettes are less harmful; (3) reducing the availability of smoked tobacco by greatly limiting retail outlets and requiring retailer licensing; and (4) increasing cessation initiatives. New Zealand’s plan will focus on severely limiting smoked products, while being less restrictive of vaping products.

A central theme of New Zealand’s Smokefree plan is its emphasis on health inequalities: the first of the three objectives is “Eliminate inequities in smoking rates and smoking-related illnesses” and the first focus area, recognizing the substantially higher burden of smoking among the Māori people, is to “Ensure Māori leadership and decision-making at all levels” [19]. Canada should be able to learn from the diverse approaches taken to endgames and endgame goals by New Zealand and other countries.

## 3. Summary and Conclusions

Although progress has been made, millions of Canadians continue to smoke, and a whole new generation risks addiction to nicotine. Tens of thousands of lives and hundreds of millions of dollars could be saved if Canada were to adopt a true Endgame strategy that disrupts the structural, political, and social dynamics that sustain the tobacco epidemic. Every year, month and day that go by without adopting such measures results in huge avoidable morbidity, mortality, human and financial cost.

A bold, reinvigorated tobacco control strategy is needed that significantly advances *ongoing* policy developments and introduces comprehensive initiatives, including key demand-reduction policies of the FCTC (WHO MPOWER measures) (e.g., a ban on advertising and promotion, increasing taxes to 70% of the retail price, and substantially improve cessation programs), as well as formidable, new *disruptive* policies and regulations. Such policies might usefully reform the processes of tobacco supply by regulating and restricting retailer activities (e.g., ending all promotion and incentive programmes, and limiting tobacco sales to tobacco-only outlets), regulating the product itself, regulating the introduction of new tobacco/nicotine product, and requiring that tobacco companies be mandated to reducing tobacco use and nicotine addiction through legislated incentives and disincentives. Canada might usefully adopt as a national policy goal, similarly to Finland, that the Endgame objective includes all nicotine use, not just tobacco use. It is important to remind ourselves that the tobacco industry and its products continue to kill more Canadians each year than have died from COVID-19 and opiate overdoses combined. The tobacco epidemic is an avoidable public-health catastrophe. Additionally, it is one which merits the development and implementation of appropriate public policies if we are to end a totally preventable pandemic of tobacco disease and death.

## Figures and Tables

**Table 1:** Four major areas for action arising from the 2016 Endgame Summit.

**1. Product Changes—Less Addictive, Less Appealing**
**2. Aligning tobacco supply with*public health* goals**— **change tobacco supply parameters:** taxation price controls reduce retail availability
**3. Prevent new generation of smokers**
**4. Transform access to cessation—no smoker left behind**

**Table 2:** Cigarette tax and price increases per province/territory, 2014–2021 (first half) inclusive.

Province or Territory	Provincial/Territorial Tax Increase	Average Manufacturing Price Increase *	Federal Tax Increase	Subtotal	Federal/Provincial/ Territorial Sales Tax Rate	Sales Tax	Total Government Tax Increases	Total Tax and Price Increases
YT	20.00	23.20	8.05	51.25	5%	2.56	30.61	53.81
NWT	3.60	23.20	8.05	34.85	5%	1.74	13.39	36.59
NU	10.00	23.20	8.05	41.25	5%	2.06	20.11	43.31
BC	14.40	23.20	8.05	45.65	5%	2.28	24.73	47.93
AB	15.00	23.20	8.05	46.25	5%	2.31	25.36	48.56
SK	4.00	23.20	8.05	35.25	11%	3.88	15.93	39.13
MB	1.00	23.20	8.05	32.25	12%	3.87	12.92	36.12
ON	12.25	23.20	8.05	43.50	13%	5.66	25.96	49.16
QC	4.00	23.20	8.05	35.25	5%	1.76	13.81	37.01
NB	13.04	23.20	8.05	44.29	15%	6.64	27.73	50.93
NS	12.00	23.20	8.05	43.25	15%	6.49	26.54	49.74
PEI	10.04	23.20	8.05	41.29	15%	6.19	24.28	47.48
NL	18.00	23.20	8.05	49.25	15%	7.39	33.44	56.64

Notes: All amounts in the table are expressed per carton of 200 cigarettes; * there is some variation in manufacturer price increases (net of tax) by province, but 2020 and 2021 breakdowns by province are not yet available; the CAD 23.20 average manufacturer price increase (net of tax) is the average Canada-wide, for 2014 to the first half of 2021 inclusive compared with 2013 (source: Health Canada based on tobacco company reports pursuant to federal *Tobacco Reporting Regulations*). Thus, the total industry average price increases per province/territory are approximate; the amount of manufacturer price increases can vary among brands; during 2014–2021, some provinces/territories have taken action to narrow the roll-your-own loophole, notably BC, MB, NS, and NU; there were no tobacco tax increases between 2 July 2021, and 1 January 2022 inclusive; there would likely be further manufacturer price increases (net of tax) in 2021 (second half) and 2022.
